# Hemangiopericytoma: Incidence, Treatment, and Prognosis Analysis Based on SEER Database

**DOI:** 10.1155/2020/2468320

**Published:** 2020-11-02

**Authors:** Kewei Wang, Fei Mei, Sisi Wu, Zui Tan

**Affiliations:** ^1^Department of Thoracic and Cardiovascular Surgery, Zhongnan Hospital of Wuhan University, Wuhan, China; ^2^Department of Vascular Surgery, The First College of Clinical Medical Science, China Three Gorges University, Yichang, China; ^3^Center of Clinical Reproductive Medicine, The First College of Clinical Medical Science, China Three Gorges University, Yichang, China

## Abstract

**Background:**

Hemangiopericytomas are rare tumors derived from pericytes surrounding the blood vessels. The clinicopathological characteristics and prognosis of hemangiopericytoma patients remain mostly unknown. In this retrospective cohort study, we assessed the clinicopathological characteristics of hemangiopericytoma patients, as well as the clinical usefulness of different treatment modalities. *Material and Methods*. We collected the clinicopathological data (between 1975 and 2016) of hemangiopericytoma and hemangioendothelioma patients from the Surveillance, Epidemiology, and End Results (SEER) database. Incidence, treatment, and patient prognosis were assessed.

**Results:**

Data from 1474 patients were analyzed in our study cohort (hemangiopericytoma: *n* = 1243; hemangioendothelioma: *n* = 231). The incidence of hemangiopericytoma in 2016 was 0.060 per 100,000 individuals. The overall survival (OS) and cancer-specific survival (CSS) did not differ between patients with hemangioendothelioma and those with hemangiopericytoma (*P* = 0.721, *P* = 0.544). The tumor grade had no effect on the OS of hemangiopericytoma patients. Multivariate analysis revealed the clinical usefulness of surgery in hemangiopericytoma patients (HR = 0.15, 95% confidence interval: 0.05-0.41, *P* < 0.001). In contrast, radiotherapy did not improve OS (*P* = 0.497) or CSS (*P* = 0.584), and chemotherapy worsened patient survival (*P* < 0.001). Additionally, the combination of surgery and radiotherapy had a similar effect with surgery alone on hemangiopericytoma patient survival (OS: *P* = 0.900; CSS: *P* = 0.156). Surgery plus chemotherapy provided a worse clinical benefit than surgery alone (*P* < 0.001).

**Conclusions:**

Our findings suggested that hemangiopericytoma had a similar prognosis with hemangioendothelioma. Surgery was the only effective treatment that provided survival benefits in hemangiopericytoma patients, while the clinical usefulness of adjuvant chemotherapy or radiotherapy was limited.

## 1. Introduction

Hemangiopericytomas were first described by Stout in 1949 as rare neoplasms arising from pericytes surrounding blood vessels [1]. Unlike other types of soft tissue tumors, the mechanisms underlying hemangiopericytoma development remain poorly understood [2, 3]. Moreover, hemangiopericytoma diagnosis and management guidelines are not well established [4, 5]. The tumor size, mitotic rates, invasiveness, and foci of hemorrhage or necrosis have been proposed as factors indicating hemangiopericytoma malignancy [6–8]. The symptoms of hemangiopericytoma can vary depending on the affected organs and tumor stage [9]. While some patients remain asymptomatic until advanced disease, the majority of patients present with pain and mass-related symptoms, including skin temperature elevation, urinary retention, and constipation. Additionally, a small proportion of hemangiopericytoma patients present with vascular disease-related symptoms. As computed tomography (CT) or magnetic resonance imaging has not been proven accurate diagnostic methods for hemangiopericytoma, hemangiopericytoma diagnosis is mostly dependent on pathological examination [10].

Currently, surgery is the standard of care for hemangiopericytoma patients. However, metastasis and tumor recurrence occur in approximately 20% of the patients [9]. Although radiotherapy may reduce the risk of local recurrence, its usefulness remains controversial [10, 11]. No guidelines regarding hemangiopericytoma chemotherapy have been established thus far. Under certain circumstances, some chemotherapeutics, including anthracycline and ifosfamide, are empirically used in clinical practice [12, 13].

The aim of this retrospective population-based study was to acquire further insight into the mechanisms underlying hemangiopericytoma development and its clinicopathological characteristics, as well as compare different treatment modality outcomes. To this end, we analyzed data from hemangiopericytoma patients using the Surveillance, Epidemiology, and End Results (SEER) database. Particularly, we evaluated hemangiopericytoma incidence, clinicopathological characteristics, treatment, and prognosis. We also compared the prognosis of patients treated with surgery, radiotherapy, or chemotherapy.

## 2. Materials and Method

### 2.1. Patient Data Acquisition

Data from patients diagnosed with hemangiopericytoma and hemangioendothelioma between 1975 and 2016 were extracted from the SEER database (Surveillance, Epidemiology, and End Results (SEER) Program (http://www.seer.cancer.gov/) SEER∗Stat Database: Incidence - SEER Research Data, 9 Registries, Nov 2019 Sub (1975-2017) - Linked To County Attributes - Time Dependent (1990-2017) Income/Rurality, 1969-2017 Counties, National Cancer Institute, DCCPS, Surveillance Research Program, released April 2020, based on the November 2019 submission). Incidence, frequency, and patient survival were analyzed for both diseases. Due to the absence of patient identifiers, approval from the Institutional Review Board was not required. Based on the International Classification of Diseases for Oncology, 3rd Edition (ICD-O-3), the histology codes 9150/3 and 9130/3 were used to identify patients with hemangiopericytoma and hemangioendothelioma, respectively. In addition, we integrated a solitary fibrous tumor (histology code 8815/3) into the analysis of hemangiopericytoma, since their combination under the common name SFT/HPC. Patients diagnosed with recurrent hemangiopericytoma or hemangioendothelioma as well as patients with nonhistologically confirmed tumors were excluded from the study.

We analyzed the clinicopathological features of hemangiopericytoma and hemangioendothelioma patients, including gender, race, marital status, age, tumor grade, tumor-node-metastasis (TNM) stage, and American Joint Committee on Cancer (AJCC) stage. The extent of hemangiopericytoma was classified as localized, regional, or metastatic as per the SEER staging criteria. We also analyzed the outcomes of anticancer interventions, including surgery, radiotherapy, and chemotherapy, for both hemangiopericytoma and hemangioendothelioma. Right censoring was performed for patients who died of other causes or were lost to follow-up.

### 2.2. Incidence and Prognosis

The incidence was reported as the rate per 100,000 individuals. The ages of all patients were adjusted to the 2000 US Standard Population standard. Additionally, to estimate patient survival, we used 1-year endpoints and calculated the annual percentage change (APC). Overall survival (OS) and cancer-specific survival (CSS) were analyzed using the Kaplan-Meier method.

### 2.3. Statistical Analysis

Disease incidence and patient survival data were acquired from the SEER database. Statistical analyses were performed using SEER∗Stat 8.3.5 software (National Cancer Institute, Bethesda, Maryland) and SPSS software (version 20.0, SPSS Inc., Chicago, IL, USA). Using 1-year endpoints in SEER∗Stat 8.3.5 software, the disease incidence and APC were calculated using a weighted-least-squares estimation. Statistical significance in parametric data was assessed using Student's *t*-test, whereas the chi-squared test was used for categorical data. Both univariate and multivariate analyses were conducted to reflect the prognostic effect of different parameters on overall survival. Specifically, the Cox proportional hazardous model was adopted for multivariate analysis. All *P* values were two-tailed. *P* values < 0.05 were considered statistically significant.

## 3. Results

### 3.1. Patient Characteristics

A total of 1474 hemangiopericytoma (*n* = 1243) or hemangioendothelioma (*n* = 231) patients were identified from 1975 to 2016. The clinicopathological characteristics of the patients are summarized in Table 1. Notably, tumors were significantly larger in hemangiopericytoma patients compared with those in hemangioendothelioma patients (median: 78 mm, mean: 97.9 mm vs. median: 33 mm, mean: 42.1 mm; *P* < 0.001). Surgery and radiotherapy were more common among hemangiopericytoma patients compared with hemangioendothelioma patients (87.7% vs. 58.9%, *P* < 0.001 and 35.9% vs. 22.5%, *P* < 0.001, respectively); however, chemotherapy was used less often (12.3% vs. 21.2%, *P* < 0.001). Besides, patients with hemangiopericytoma were significantly older than patients with hemangioendothelioma (median: 57, mean: 55.1 vs. median: 50, mean: 49.3; *P* < 0.001). No significant differences were observed in gender or ethnicity between the two groups.

### 3.2. Tumor Characteristics

Tumor characteristics, including TNM, SEER, and AJCC stages, are summarized in Table 2. The majority of patients were diagnosed with T2 tumors (70.6%), followed by T1 (20.6%). N0 and N1 stages accounted for 90.2% and 1.1% of all tumors, respectively. Metastatic disease (M1) was diagnosed in 10.4% of cases, while 89.6% of the patients had no distant metastasis at the time of diagnosis. Regarding SEER staging, 16.9% and 9.8% of patients had regional or distant metastasis, respectively. Furthermore, most patients had AJCC stage I cancer (55.2%), followed by stage III (19.8%). Regarding tumor differentiation, 14.9%, 3.9%, 9%, and 14.3% of the tumors were classified as grade I, II, III, or IV, respectively.

### 3.3. Hemangiopericytoma and Hemangioendothelioma Incidence

**Figure 1:**
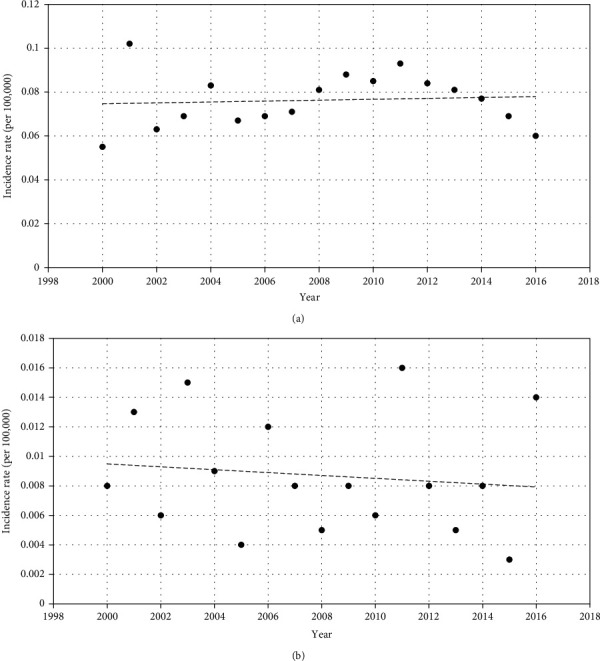
(a, b) Incidence of hemangiopericytoma (a) and hemangioendothelioma (b). Rates per 100,000 individuals are provided, and patient ages are adjusted as per the 2000 US Standard Population standard.

The age-adjusted incidence rates of hemangiopericytoma and hemangioendothelioma in 2016 were 0.060 and 0.014, respectively (Table 1). The incidence of both hemangiopericytoma (APC: 0.023; *P* = 0.98) and hemangioendothelioma (APC: -0.259; *P* = 0.91) remained steady between 2000 and 2016 (Table 1; Figure 1).

### 3.4. Treatment Outcomes

The treatment interventions provided in patients with hemangiopericytoma are summarized in Table 3. Among all treatments, only surgery provided a significant clinical benefit for hemangiopericytoma patients (univariate analysis: HR = 0.38, *P* < 0.001; multivariate analysis: HR = 0.15, *P* = 0.014). Univariate analysis revealed that chemotherapy could be detrimental (HR = 1.88, *P* < 0.001), but multivariate analysis indicated this effect to be insignificant (HR = 1.73, *P* = 0.235). Radiotherapy showed no significant effect on the OS of hemangiopericytoma patients (univariate analysis: HR = 0.96, *P* = 0.608).

**Figure 2:**
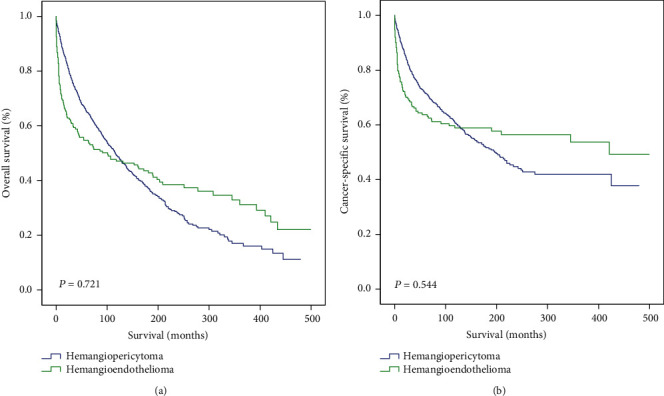
(a, b) Overall survival (a) and cancer-specific survival (b) of hemangiopericytoma and hemangioendothelioma patients. *P* values < 0.05 were considered statistically significant.

The tumor grade did not influence the OS of hemangiopericytoma patients. Notably, the OS of hemangiopericytoma patients with regional metastasis, according to the SEER staging system, did not differ significantly compared with patients with localized tumors. Similarly, no difference in OS was observed among hemangiopericytoma patients with tumors of different AJCC stages. Besides, age could serve as a protective factor for OS in patients with hemangiopericytoma (HR = 1.04, *P* = 0.002), indicated by multivariate analysis. Furthermore, gender was not a significant prognostic factor for OS in patients with hemangiopericytoma. Additionally, no significant differences in OS (*P* = 0.721) or CSS (*P* = 0.544) were observed between hemangiopericytoma and hemangioendothelioma patients (Figure 2).

**Figure 3:**
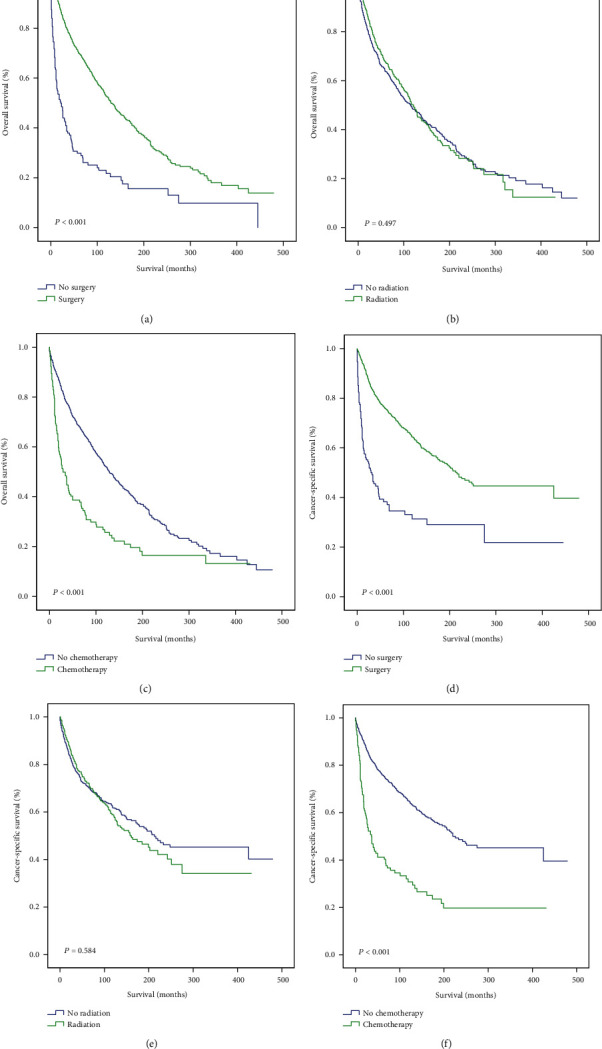
Outcomes of different treatments in hemangiopericytoma patients. OS (a–c) and CSS (d–f) of hemangiopericytoma patients treated with surgery vs. no surgery (a, d), radiotherapy vs. no radiotherapy (b, e), and chemotherapy vs. no chemotherapy (c, f). OS: overall survival; CSS: cancer-specific survival. *P* values < 0.05 were considered statistically significant.

Next, we conducted a survival analysis to evaluate the outcomes of different treatment strategies. Surgery provided a significant clinical benefit in both hemangiopericytoma (*P* < 0.001 for both OS and CSS) and hemangioendothelioma (*P* < 0.001 for both OS and CSS) patients (Figures 3(a) and 3(d); Supplementary Figures 1A and 1D). In contrast, radiotherapy did not improve survival in patients with hemangiopericytoma (OS: *P* = 0.497; CSS: *P* = 0.584) or hemangioendothelioma (OS: *P* = 0.457; CSS: *P* = 0.260) (Figures 3(b) and 3(e); Supplementary Figures 1B and 1E). Interestingly, chemotherapy significantly impaired OS (*P* < 0.001) and CSS (*P* < 0.001) in hemangiopericytoma patients (Figures 3(c) and 3(f)). In hemangioendothelioma patients, although chemotherapy decreased CSS (*P* = 0.045), it had no effect on OS (*P* = 0.209; Supplementary Figures 1C and 1F).

**Figure 4:**
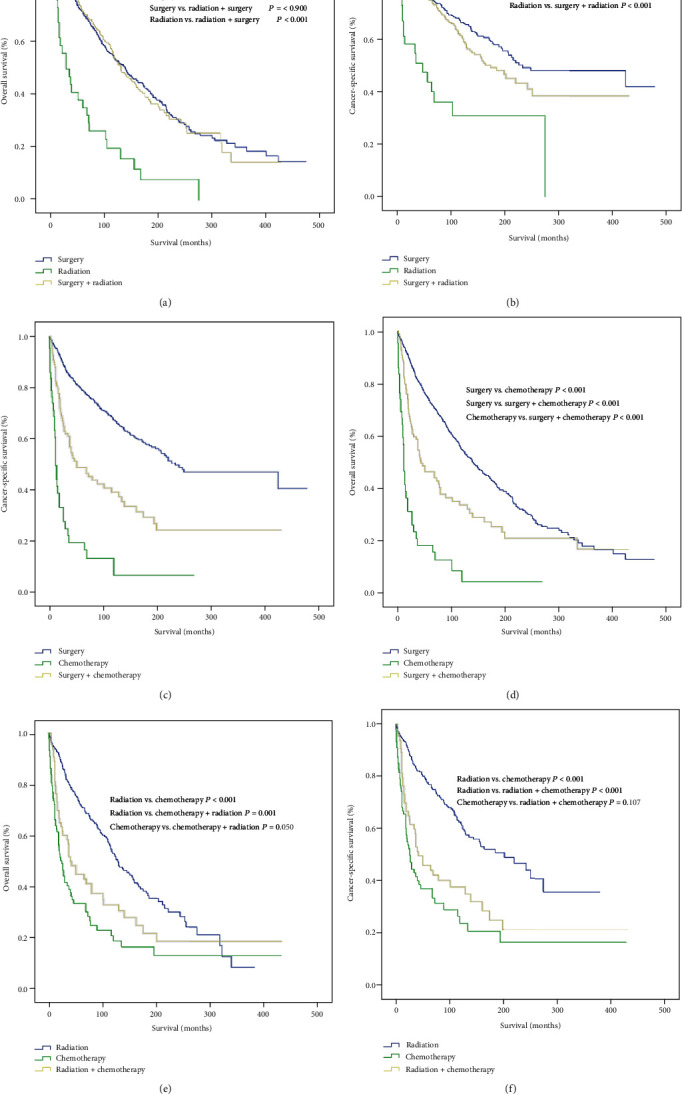
Outcomes of different monotherapies and combination therapies in hemangiopericytoma patients. OS and CSS of patients treated with surgery, radiotherapy, and surgery plus radiotherapy (a, b); surgery, chemotherapy, and surgery plus chemotherapy (c, d); radiotherapy, chemotherapy, and radiotherapy plus chemotherapy (e, f). OS: overall survival; CSS: cancer-specific survival. *P* values < 0.05 were considered statistically significant.

We also assessed the effects of different monotherapies or combination therapies on the survival of hemangiopericytoma patients. Surgery was superior in improving OS and CSS in hemangiopericytoma patients (Figures 4(a)–4(d)), followed by radiotherapy and chemotherapy (Figures 4(e) and 4(f)). Additionally, surgery plus radiotherapy was more advantageous than radiotherapy alone in terms of patient survival and had a similar effect with surgery alone (Figure 4(a) and (Figure 4(b)). Surgery plus chemotherapy improved survival to a greater extent than chemotherapy alone, although the outcomes of surgery alone were more favorable (Figures 4(c) and 4(d)). Radiotherapy plus chemotherapy was superior compared with chemotherapy alone; however, the outcomes of radiotherapy plus chemotherapy were worse than those of radiotherapy alone (Figures 4(e) and 4(f)).

## 4. Discussion

Hemangiopericytomas are rare tumors derived from pericytes surrounding the blood vessels and capillaries [1]. Hemangiopericytomas are more common among middle-aged individuals than in infants and children [14]. In this study, we found no significant differences in gender or race, between hemangiopericytoma and hemangioendothelioma patients (Table 1). The incidence of both hemangioendothelioma and hemangiopericytoma remained stable between 2000 and 2016.

Hemangiopericytomas often affect the lower extremities, retroperitoneum, pelvis, meninges, lungs, and pleura [6, 15] while less frequently affect the breast, bones, liver, pancreas, stomach, ovary, and vagina [9]. Patients with hemangiopericytoma may remain asymptomatic until advanced disease, primarily due to the indolent behavior of these tumors. However, in this study, we found that the majority of hemangiopericytoma patients were diagnosed with early-stage tumors.

The indolent behavior of hemangiopericytomas magnifies the necessity of early diagnosis, which is often based on pathological examination or imaging modalities to a lesser extent [16]. Radiographic findings include round masses with homogenous density and sharp margins, while enhanced CT can detect circumscribed masses characterized by tissue necrosis or calcification in some cases [15]. The masses often displace neighboring organs, such as the bladder, ureters, and colon, which may lead to the development of symptoms. However, a biopsy or tumor resection is required to confirm hemangiopericytoma diagnosis, which could be misdiagnosed as a different type of soft tissue tumor. Pericytes lack typical characteristics under a light microscope. Moreover, the histopathological diagnosis of hemangiopericytoma heavily relies on the presence of vessel branching [15]. Hence, in clinical practice, hemangiopericytoma is often diagnosed by exclusion. Immunohistochemical staining for vimentin and collagen type IV has also been proposed as a method to confirm hemangiopericytoma, in combination with negative stain for S-100, desmin, laminin, cytokeratins, and factor VIII-related antigen. Recently, positive immunostaining of STAT6 was also reported to have strong diagnostic value for hemangiopericytoma [17]. At the genetic level, the fusion of NAB2-STAT6, also a subtype classification of hemangiopericytoma, was observed in a great proportion of patients [4]. Furthermore, the expression of vascular endothelial growth factor receptor (VEGFR) is elevated in hemangiopericytomas; thus, VEGF-VEGFR pathway activation could serve as a diagnostic marker for hemangiopericytoma [12, 18].

To date, the definition of malignant hemangiopericytoma remains controversial [19]. The combination of a high mitotic index, large tumor size, high degree of cellularity, presence of immature tumor cells, and presence of hemorrhagic or necrotic foci has been proposed to define the malignancy of hemangiopericytoma [7, 15]. Additionally, as the incidence of metastasis varies immensely among hemangiopericytoma patients, tumor invasiveness has also been proposed as an indicator of hemangiopericytoma malignancy [6]. The prognosis and clinical characteristics of hemangiopericytoma patients also vary greatly. In this study, we found no significant differences in OS and CSS between hemangioendothelioma and hemangiopericytoma patients. Hemangioendotheliomas are intermediate-grade malignancies derived from blood vessels. Hemangioendothelioma subtypes include epithelioid hemangioendothelioma, Kaposiform hemangioendothelioma, hobnail hemangioendothelioma, and polymorphous hemangioendothelioma [20–22]. Due to their clinicopathological characteristics, hemangioendotheliomas are classified between hemangiomas (benign) and angiosarcomas (malignant). The finding that the majority of hemangiopericytoma patients were diagnosed at an early stage suggests that hemangiopericytomas may represent intermediate-grade malignancies, similar to hemangioendothelioma.

Surgery remains the standard of care for hemangiopericytoma patients. As expected, surgery considerably improved OS and CSS in hemangiopericytoma patients. However, the risk of recurrence after surgery should not be neglected, as more than 30% of hemangiopericytoma patients experience tumor recurrence after surgery [12]. Notably, common recurrence sites include the retroperitoneum and pelvis. In contrast to surgery, radiotherapy did not provide a significant survival benefit in hemangiopericytoma or hemangioendothelioma patients. Consistently, the findings of a previous study led to the speculation that hemangiopericytomas are radioresistant [23]. However, radiotherapy is considered to reduce the risk of local recurrence in soft tissue malignancies; hence, it is recommended for hemangiopericytoma patients with tumors > 5 cm or inadequate resection margins [11, 24].

The clinical benefit of chemotherapy in patients with soft tissue tumors remains unclear. Previous studies suggested that chemotherapy could be effective for patients with metastatic hemangiopericytoma [23, 25]. Importantly, the use of adriamycin was associated with disease remission in approximately 50% of patients [23]. The use of temozolomide and bevacizumab in metastatic hemangiopericytoma patients resulted in a median progression-free survival of 17 months and an overall response rate of 21.4%. Antiangiogenic reagents were also reported to provide a clinical benefit in hemangiopericytoma patients [9]. Our findings suggested that chemotherapy could even be detrimental for hemangiopericytoma patients. Future studies in large cohorts are required to further elucidate the clinical usefulness of chemotherapy in hemangiopericytoma.

Interestingly, adjuvant chemotherapy or radiotherapy combined with surgery did not improve outcomes compared with surgery alone. Notably, the combination of surgery with chemotherapy worsened OS and CSS in hemangiopericytoma patients. Hence, we believe that surgery should be preferred to other therapeutic modalities and that adjuvant chemotherapy or radiotherapy may not be as promising as previously considered [12, 16]. Future large multicenter studies are required to further assess the clinical usefulness of chemotherapy and radiotherapy in hemangiopericytoma.

There were several limitations to this study. First, our findings and conclusions were solely based on analyses from data acquired from the SEER database. Second, details on treatments, including surgery type, medical prescriptions, and radiotherapy protocols, could not be accessed, restricting our analyses. Third, biases could have been introduced due to the retrospective nature of the study. Nevertheless, data mining from SEER and other databases can help minimize biases of analysis caused by different institutions. Additionally, compared with case reports, the comprehensive clinicopathological characteristics and relatively large patient cohort can be more informative in analyzing hemangiopericytomas and other rare tumors.

## 5. Conclusions

The findings of this population-based study confirmed the low incidence of hemangiopericytoma and highlighted that hemangioendothelioma and hemangiopericytoma had a similar prognosis. Surgery remains the only effective treatment that can provide significant survival benefits in hemangiopericytoma patients, while the clinical usefulness of adjuvant chemotherapy or radiotherapy is limited.

## Figures and Tables

**Table 1:** Clinicopathological characteristics of hemangiopericytoma and hemangioendothelioma patients.

Factor, *n* (%)		Hemangiopericytoma (*n* = 1243)	Hemangioendothelioma (*n* = 231)	*P*
Year (%)	1975-1986	118 (9.5)	86 (37.2)	<0.001
	1987-1996	146 (11.7)	38 (16.5)	
	1997-2006	392 (31.5)	48 (20.8)	
	2007-2016	587 (47.2)	59 (25.5)	
Age (years)	Median	57	50	<0.001
	Mean	55.1	49.3	
	Range	0-98	0-97	
Size (mm)	Median	78	33	<0.001
	Mean	97.9	42.1	
	Range	0-989	3-190	
Sex (%)	Male	583 (46.9)	120 (51.9)	0.159
	Female	660 (53.1)	111 (48.1)	
Race/ethnicity (%)	White	1000 (81.3)	180 (77.9)	0.731
	Black	114 (9.3)	22 (9.5)	
	Other	116 (9.4)	29 (12.5)	
Surgery (%)	Yes	1090 (87.7)	136 (58.9)	<0.001
	No	153 (12.3)	95 (41.1)	
Chemotherapy (%)	Yes	153 (12.3)	49 (21.2)	<0.001
	No	1090 (87.7)	182 (78.8)	
Radiotherapy (%)	Yes	442 (35.9)	52 (22.5)	<0.001
	No	788 (64.1)	179 (77.5)	
Incidence (2016)		0.060	0.014	
Annual percentage change (2000-2016)		0.023	-0.259	
		*P* = 0.98	*P* = 0.91	

*P* value less than 0.05 is statistically significant.

**Table 2:** TNM and SEER stages in the hemangiopericytoma patient cohort.

Hemangiopericytoma (AJCC stage 7th edition)					
	Number	Percent (%)		Number	Percent (%)
T stage			SEER stage		
T1	35	20.6	Localized	442	37.8
T2	120	70.6	Regional	197	16.9
Unknown	15	8.8	Distant	115	9.8
N stage			Unstaged	415	34.7
N0	165	90.2	Grade		
N1	2	1.1	I	142	14.9
Unknown	16	8.7	II	37	3.9
M stage			III	86	9.0
M0	164	89.6	IV	136	14.3
M1	19	10.4	Unknown	552	57.9
AJCC stage					
I	95	55.2			
II	17	9.9			
III	34	19.8			
IV	19	11.0			
Unknown	7	4.1			

TNM: tumor-node-metastasis; SEER: Surveillance, Epidemiology, and End Results; AJCC: American Joint Committee on Cancer.

**Table 3:** Univariate and multivariate analyses for the overall survival of hemangiopericytoma patients.

Parameter	Univariate analysis		Multivariate analysis	
	*P*	HR (95% CI)	*P*	HR (95% CI)
Age	<0.001	1.04 (1.03-1.04)	0.002	1.04 (1.01-1.06)
Gender				
Female	Reference			
Male	0.369	0.94 (0.81-1.08)		
Surgery				
No	Reference		Reference	
Yes	<0.001	0.38 (0.32-0.45)	<0.001	0.15 (0.05-0.41)
Chemotherapy				
No	Reference		Reference	
Yes	<0.001	1.88 (1.56-2.25)	0.235	1.73 (0.70-4.25)
Radiotherapy				
No	Reference			
Yes	0.608	0.96 (0.83-1.12)		
Grade				
Unknown	Reference		Reference	
Well differentiated	<0.001	0.60 (0.48-0.76)	0.622	0.71 (0.18-2.76)
Moderately differentiated	0.601	0.86 (0.48-1.53)	0.122	3.50 (0.72-17.08)
Poorly differentiated	0.139	1.23 (0.93-1.63)	0.034	3.53 (1.10-11.39)
Undifferentiated	0.044	1.29 (1.01-1.64)	0.069	2.86 (0.92-8.85)
SEER historic stage				
Localized	Reference		Reference	
Regional	<0.001	1.49 (1.20-1.86)	0.022	2.59 (1.15-5.82)
Distant	<0.001	3.70 (2.98-4.61)	—	—
Unstaged	<0.001	1.57 (1.31-1.87)	0.229	0.25 (0.03-2.37)
AJCC stage				
Unknown	Reference		Reference	
I	0.621	0.74 (0.22-2.50)	0.125	0.25 (0.05-1.48)
II	0.952	1.05 (0.23-4.69)	0.292	0.32 (0.04-2.67)
III	0.055	3.28 (0.98-11.02)	0.474	0.49 (0.69-3.46)
IV	0.008	5.37 (1.56-18.49)	—	—

AJCC: American Joint Committee on Cancer; HR: hazard ratio; CI: confidence interval. *P* < 0.05 was considered to be significant.

## Data Availability

The data we used in this study can be downloaded from the SEER (Surveillance, Epidemiology, and End Results Program, 1975-2016) database.

## Abbreviations

AJCC: American Joint Committee on Cancer
CSS: Cancer-specific survival
OS: Overall survival
SEER: Surveillance, Epidemiology, and End Results
TNM: Tumor-node-metastasis.

## Supplementary Materials

Supplementary Materials Supplementary Figure 1; outcomes of different treatments in hemangioendothelioma patients. OS (A-C) and CSS (D-F) of hemangioendothelioma patients treated with surgery vs. no surgery (A, D), radiotherapy vs. no radiotherapy (B, E), and chemotherapy vs. no chemotherapy (C, F). OS: overall survival; CSS: cancer-specific survival. *P* values < 0.05 were considered statistically significant.
