# Supplementary material for: Hemangiopericytoma: Incidence, Treatment, and Prognosis Analysis Based on SEER Database
Source: Biomed Res Int. 2020 Nov 2;2020:2468320. doi: 10.1155/2020/2468320 (PMC7655240; doi:10.1155/2020/2468320)
Supplement: Supplementary Materials — Supplementary Figure 1; outcomes of different treatments in hemangioendothelioma patients. OS (A-C) and CSS (D-F) of hemangioendothelioma patients treated with surgery vs. no surgery (A, D), radiotherapy vs. no radiotherapy (B, E), and chemotherapy vs. no chemotherapy (C, F). OS: overall survival; CSS: cancer-specific survival. P values < 0.05 were considered statistically significant. [file 2468320.f1.docx]

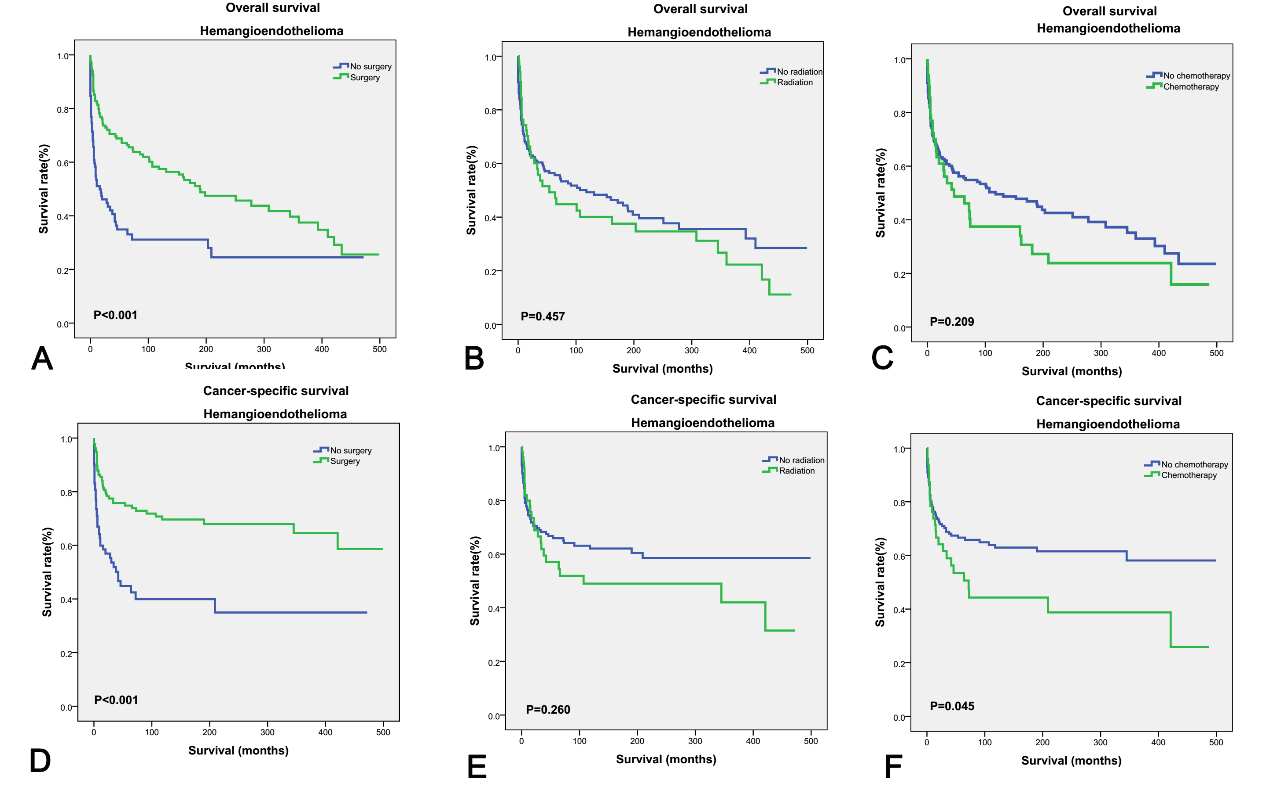


**Supplementary Figure 1.** Outcomes of different treatments in hemangioendothelioma patients. OS (A-C) and CSS (D-F) of hemangioendothelioma patients treated with surgery vs. no surgery (A, D), radiotherapy vs. no radiotherapy (B, E), and chemotherapy vs. no chemotherapy (C, F). OS, overall survival; CSS, cancer-specific survival. P-values <0.05 were considered statistically significant.
